# Hospital and Institutional News

**Published:** 1920-04-03

**Authors:** 


### HOSPITAL AND INSTITUTIONAL NEWS.

### THE PRINCE OF WALES AND THE ITALIAN HOSPITAL.

The Prince of Wales has become a patron of the Italian Hospital, in Queen Square, which is seeking to commemorate the work done by the hospital during the war by the endowment of three beds. These will be called respectively the British, the Colonial, and the Italian bed. The last is to be endowed by Miss Cerasoli. The hospital is considering whether or not to organise contributions from patients.

### THE MEDICAL RESEARCH COMMITTEE.

On April 1 the title of the Medical Research Committee constituted by the National Health Insurance Regulations of 1914 ceased to exist. Instead the members and various workers included in this body become a Medical Research Council, carrying out their work as before, but acting under the direction of a Committee of the Privy Council. This Committee consists of the Lord President of the Council, the Minister of Health, the Secretary for Scotland, and the Chief Secretary for Ireland.

### THE ASSOCIATION OF SURGEONS.

The first meeting of the newly-formed Association of Surgeons of Great Britain and Ireland will be held in London in May. It is but a short time since we had pleasure in welcoming the formation of this professional body, and, as was then pointed out, the development represents a complete break from the conservative traditions of the past and brings together the best surgical minds in the country for purposes of mutual interest and invaluable scientific advancement. Similar organisations have been long established in a number of other countries, and have, we believe, met everywhere with unqualified success.

### SPLENDID GIFT TO SWANSEA HOSPITAL.

A munificent gift to the Swansea Hospital was announced on March 25. The Rev. H. C. Mander reported that the pressure on the beds would be greatly relieved by the action of Mr. Roger Beck, who had that afternoon undertaken to purchase Parc Wern House, Swansea, for £16,000, and would make a present of it to the hospital on the condition that it should be used as an adjunct of the General Hospital. The grounds of the house comprise about 18 acres. Mr. Beck has done great things for the Swansea Hospital. Among his other benefactions was the endowment of a bed to "Marcus Beck, M.P.,"; the endowment of two beds in the Beck Ward, and a contribution of £1,000 at the time of the Armistice Appeal. Mr. Beck, replying to a vote of thanks, acknowledged that he owed a great deal to Labour in his daily business, and it was in recognition of what Labour had done for him that he had purchased this estate. He felt he could not do better than make this presentation to the institution in which Labour had such an interest. Another anonymous gift of £1,000 has also been received.

### SHORTAGE OF DENTAL SURGEONS.

There is some satisfaction, when one reads the recent statement of the Committee of Management of the Royal Dental Hospital, in noting the greatly increased number of students now attending the dental schools of the country. The supply of properly qualified dental practitioners is still quite inadequate to the needs of the nation, for at present there are only somewhere about 4,500 duly qualified men on the dental register. It is good to learn, however, that an increasing number of women are training for this profession, for there is an insistent and obvious demand for more minute dental care of multitudes of school children, the treatment of whom could be accomplished on far more comprehensive lines if more women dentists were available. At the annual meeting of the Royal Dental Hospital, where many of these questions came under notice, Sir Fisher Dilke, presiding, announced that the Prince of Wales had consented to be the President of the hospital.

### A HOPEFUL OUTLOOK AT LEEDS.

Notwithstanding the financial difficulties, Mr. Charles Lupton takes a hopeful view of the future of the Leeds Infirmary. Speaking at the annual meeting, he said that the Workpeople's Hospital Fund Committee had decided to contribute £20,000 a year instead of £10,000. From Mr. Braime's scheme for employers' subscriptions, already described in full in The Hospital, it is hoped to receive from £10,000 to £15,000 a year. He recorded also Mr. Joseph Watson's princely gift of £50,000. Mr. Lupton also well said that all his reasons for hope "do not appear in the account," and this seems to emphasise the fact that hospital accounts cannot be judged as are the balance sheets of business, a fact utterly disregarded by the London newspapers. Mr. Lupton, who gave a comprehensive survey of the hospital problems of Leeds, deprecated the establishment of a separate children's hospital. One in every six of the new out-patients admitted last year was a child. He wished to increase the accommodation for children, and to group them apart in the hospital, to keep the accounts separate, and to allow subscriptions to be earmarked for the work. The board wanted to establish and endow another wing at its semi-convalescent hospitals for tuberculous children, where they could be kept for sixteen months, but for ten years he had waited for its endowment. With men like Mr. Lupton at the helm, and with the methods suggested by Mr. Braime, it is impossible to doubt the resources of the voluntary system.

### ANOTHER PAY HOSPITAL FOR LONDON.

The new Freemasons' Hospital and Nursing Home will be opened in May on the premises formerly occupied by the Chelsea Hospital for Women. The institution is intended for those members of the Craft who are not in a financial position to go into an ordinary nursing home and pay the charges together with the medical and surgical fees involved.

The idea is to establish an intermediate institution where the patients are charged what is the cost today of a bed in any ordinary public hospital. At the same time patients will be encouraged to pay some nominal charge towards the surgical expenses and those of the anæsthetist. The electrical and *x*-ray department will be a feature of the institution, which is probably as well equipped as any hospital of its size in the country. The hospital will take a limited number of women and children over twelve years of age, in addition to the male patients. No infectious cases and no chronic cases will be admitted.

### THE BRISTOL VOLUNTARY LOCK HOSPITAL.

In view of the establishment of clinics for venereal disease, the Bristol Voluntary Lock Hospital, in Ashley Road, has been considering whether to continue its fifty years' record of work. It is a small homelike institution where patients, who are women only, can be cared for before treatment and trained for respectable work during recovery. At a recent meeting of the medical officers of the V.D. clinics it was resolved that the hospital should continue because it provides for a class of patient not otherwise received. The treatment is to be modified, but the medical officers believe that the hospital is filling an important need in the moral training which it gives to the patients.

### THE HOSPITAL IN HARROW ROAD.

The London Lock Hospital, which has a male branch in Dean Street, Soho, and a female branch in Harrow Road, is busy with schemes for extension. The report states that no other institution provides up-to-date residential accommodation for maternity cases suffering from venereal disease, and that patients come from every part of the United Kingdom. The plans, which have been approved by the King's Fund, comprise a new wing with eighty-six beds on three floors, and £80,000 is wanted. It will be circulated everywhere because the patients come from every part. It is hoped to raise the above sum by the end of the year.

### THE GALLIO OF COMMERCE.

We do not wish to mark any one class for censure in its relation to the voluntary hospitals, but the fact remains that in industry the retail tradesmen seem to be the most indifferent section of all. The only shop which normally has an organised system of subscription to hospitals is the workshop, or, in other words, the factory. The retail shopman, like a commercial Gallio, seems to care for none of these things. Evidence in support of this contention is far too common. It is found all over the country. Thus at Redditch, where a collection was recently held, we learn that "nearly every factory in the town subscribed, but the result of the special appeal to the tradespeople was very disappointing." The belief that retail trade is bad for the character is as old as Aristotle, and would not have endured if there was no reason for it. People whose income depends on a penny made here and sixpence made there can hardly escape from the fate of becoming small-minded. They are subdued to what they work in, and it would be at least poetically just if the fines imposed for profiteering by tradesmen could be handed to their local hospital. We should like to see an attempt made to interest the retailers in London and other towns. Where the employees and employers have led, the tradespeople should be induced to follow.

### £100,000 FOR AMALGAMATION AT BRISTOL.

At a meeting in aid of the children of Central Europe the Lord Mayor of Bristol made a remarkable announcement. "I had a gentleman here the other day in connection with our hospitals," he said, "who is willing to give £100,000 himself if I can only get the hospitals of this city to amalgamate under one committee. That sum would bring £5,200 a year." As we have already reported, the case for amalgamation has been actively discussed in Bristol, and though the objections are more serious than the supporters of the plan seem to realise, there is no reason why the proposal should not be tried. One experiment of this kind, whatever result it produced, would be an object-lesson; and if it is to be tried at all it had better be tried where it is most favoured and receives the strongest backing.

### THE EXTENSION OF HIGHBURY.

The important extensions required at the Highbury and Uffculme hospitals for ex-Serivce men of the West Midland area will enable 320 men eventually to be treated. There is abundant need for them and much local anxiety over the condition of some of the men upon their demobilisation. The Treasury has granted £25,000 towards the extensions; and the Ministry of Pensions will bear the cost of maintenance.

### BIDEFORD AND BRAINS.

The Bideford and District General Hospital has had a very successful year, a year in which practically every source of income showed an increase. When we examine the reason for this satisfactory condition of affairs it appears to be due to the personal service of a few collectors, who not only went their rounds, so to speak, as usual, but were alert to put forward the hospital's claim to any balance of funds remaining to local war organisations. With such helpers Mr. John Lamerton, the secretary, should have good hopes of completing the extension fund. A hospital not in financial straits sets a good example. It proves what can be done when the intelligence is there to do it.

### A GUARDIAN'S HOSPITAL AT BARNET.

The Barnet Board of Guardians has decided to open the Union hospital buildings as a hospital available for all patients in the Union area on the recommendation of the medical officer. There will be no fixed charge, but patients will pay according to their means. During the war the buildings were occupied by the military, with whom protracted negotiations are in progress. The plan will be watched with interest, for there are fourteen parishes in the Barnet Union, with a population of 100,000, and the Victoria Hospital has more work than it can do even in the Barnet district.

### PENSION FOR A HOSPITAL CHAPLAIN.

The Rev. M. T. Friend, who retired from the post of Chaplain to the Royal Berkshire Hospital, Reading, in December, has been granted at the recent annual court a retiring pension of £100 per annum. Mr. Friend has a record of over thirty-seven years' service. He is succeeded by the Rev. F. J. C. Gillmor, in whose parish the hospital stands. It is not, therefore, to be a whole-time appointment. We are glad to record this recognition of a long period of service, and congratulate Mr. Friend and the Committee on the work and its appreciation.

### CHEERING NEWS FROM CHESTERFIELD.

A very successful financial year has been completed by the Chesterfield and North Derbyshire Hospital, which, with an expenditure approaching £17,000, was only £53 to the bad. About half the income resulted from workmen's subscriptions. The hospital's future is full of promise. A proposal is favoured to co-ordinate with it the work of the proposed maternity hospital. The gift of Holywell House and grounds by Alderman Eastwood, the chairman of the ward, is invaluable because it is the only site where extensions can be made. A new out-patient department is wanted; more room for the staff, which will have to be increased when the working hours are reduced, is also desired. A laundry is also under consideration. Altogether the report is one of the best yet presented, and may well cheer other hospitals upon their way.

### PAYING PATIENTS IN COTTAGE HOSPITALS.

To increase its income so as to cover the present cost of maintenance, the committee of the Victoria Cottage Hospital, Romford, has revised its procedure in respect of paying patients. The admission of patients at normal fees shall not exceed half the total accommodation, so that those able to pay reduced fees, or those admitted free, shall have a fair share of accommodation. Patients are to pay in accordance with their means, and the fees are to be entered on the application form so that each patient may know the payments in advance. Members of associations, societies, or employees of works which subscribe to the hospital shall be admitted free, but where the body corporate makes financial arrangements with a doctor particulars must be forwarded to the hospital. In short, the principle of payment according to means has been established and will be applied more methodically than hitherto.

### SMOKING: AN EXPERIMENT.

Some time ago it was suggested that the patients at King Edward VII. Hospital, Cardiff, should be allowed to smoke at specified times or under specified conditions. The matter was referred to the Medical Board, and at the last meeting of the Board of Management it was decided, upon the recommendation of the Medical Board, that smoking should be permitted in the wards between the hours of 5 p.m. and 7 p.m., as a tentative measure. The interests of patients to whom smoking may be prejudicial or offensive are to be safeguarded, and the permission is experimental for three months. It would be interesting to know if such permission is given in any other hospital. We do not feel quite sure that fire insurance companies will not raise their premiums where this experiment is imitated, and, on the whole, they would probably be wise to do so.

### PUBLIC BATH IN A HOSPITAL.

As a curious commentary on the habits of sixty years ago, Mr. Rea, in a recent address, quoted from an advertisement which appears on the fly-leaf of the King Edward VII. Hospital, Cardiff, report for 1862—"Rule 27. That persons who are not patients at the infirmary may use the lower bath (which is conveniently situated for general use, and will be reserved entirely for the public) upon payment of 1s., for a warm or cold bath, or £2 2s. per quarter." Some years ago he had pointed this out to an elderly member of the Committee, since dead, who well remembered the conditions, and even earlier, when the house surgeon for the time being was obliged to repair to a neighbouring hotel for his periodic bath.

### A FINE RECORD OF PERSONAL SERVICE.

Mostly made up of coppers and, perhaps, an occasional silver coin, a lady at Brighton has just completed the collection of £1,000, which sum week by week has gone to the funds of the Royal Sussex County Hospital. It has taken some years to accomplish, but the governors have just placed it on record. On visiting days Miss Patching or her deputy has posted herself inside the entrance to the institution, and as the visitors leave they have been invited to place any small sum in her collecting-box. No one, because this excellent labour of love is being continued, is ever pressed, but the reminder has been made so quietly that visitors have felt constrained to respond. The governors have described Miss Patching's work as "truly a fine record," and one may add that it proves the truth of our persistent contention that personal service works wonders, even by the smallest means.

### PUBLICANS AND DELIRIUM TREMENS.

A strange request has been made to the boards of guardians in the district of South London over which the Licensing Justices of the Newington Division have jurisdiction. The Justices desire to be informed of all cases of licensees of public houses suffering from delirum tremens who come under the notice of the guardians, as they are of opinion that a licensee suffering from such a complaint is not a fit and proper person to hold a licence. In most cases where this request has been made the guardians have refused to give the information, holding that to comply with it would involve a departure from their practice not to divulge particulars relating to the nature of a complaint from which a person in in their care may be suffering, unless under legal process. They are trying to follow the practice of the medical profession, which has always declined to give similar information.

### WAS BUNYAN MAD?

IN a paper read before the Royal Society of Arts the Rev. E. G. O'Donoghue, the chaplain of Bethlem, said that apparently nobody realised that for three or four years of his life Bunyan was insane, in the technical and medical sense of the word. The lecturer was of opinion that the book "Grace Abounding" contains particulars sufficient to fill up the certificate and case-book of the mental specialist. Premonitory symptoms of the latent disease manifested themselves from time to time, and in 1651 the "great storm came stealing" upon him, and "floods of blasphemies," unprovoked and unexpected, overwhelmed his mind. Like other victims of religious melancholia, he added to his physical agitation and mental agony by ransacking the Scriptures to justify the appalling sentence of everlasting damnation that he was convinced had been pronounced against his soul. But John Bunyan won through, and subsequently produced his greatest work, "Pilgrim's Progress."

### A DIPLOMA OF TUBERCULOSIS.

THE health committee of the Deptford Borough Council feels that the status of tuberculosis officers would be greatly improved if examination boards were empowered to grant a diploma in pulmonary, surgical, and general tuberculosis. The committee accordingly suggests that the Ministry of Health should give favourable consideration to this proposal.

### THE REGULATION OF VISITORS.

THE medical superintendent of the Camberwell Infirmary reports to the Board of Guardians that more visitors come than is good for the patients and that they stay much too late. He proposes that the ordinary cards for those on the "Danger" list should state that visiting is permitted between 2 and 9, and that the wards are closed from 4.30 to 5.15 and from 7.30 to 8. A limited number of patients will be placed on the "Full Danger" list and the visitors to those patients will be permitted to come in at any time during the day, subject to the requirements of the nursing, etc., and to stay the night with special permission. The card, which must be shown at the gate, is only available for two visitors at a time. In addition to the "Danger" list cards, he is having "Special" permits printed for the benefit of those whose work does not allow them to come on visiting days.

### HOW SHOULD SUNDAY FUND GRANTS BE DETERMINED.

THE Melbourne Hospital Saturday and Sunday Fund Committee has made its annual grants to the metropolitan medical charities, which alone are entitled to participate in the Fund. The distribution of this fund is based, in the case of the hospitals, on their maintenance expenditure for the previous year, irrespective of the number of occupied beds. The system, writes our Australian correspondent, is not universally approved, and objectors seem to regard it as a premium on extravagance. When all the claims made on the charitable during the war are remembered, the amount contributed of late years to this Fund has been remarkable, being from four to five times as much as was collected at the early stages of the movement.

### TRAFFIC AND NURSING HOMES.

MARYLEBONE BOROUGH COUNCIL has recently received several complaints with regard to the vibration and serious inconvenience caused by the increasing number of heavy motor-lorries, traction-engines and their trucks which travel through residential streets, including those which contain many nursing homes. It is urged that the vibration from this heavy traffic is not only detrimental to property, but that the noise and smoke from the engines contribute a public nuisance. The Borough Council has drawn the attention of the Ministry of Transport to the complaints, and suggested that residential streets, especially those in which there are nursing homes, should not bear too large a proportion of the heavy traffic complained of. Can certain streets be forbidden to these lorries, and, if so, how?

### A REBATE ON HOSPITAL SUBSCRIPTIONS?

THE Committee of the Birmingham and Midland Eye Hospital has received an offer from Messrs. Cadbury Brothers to pay the cost of in-patient treatment of any of their employees and dependants. The cost is to be that of the previous financial year. It is hoped that other firms will follow suit. The Committee also makes a suggestion to the public. "As is well known," the report proceeds, "a firm's subscriptions to local hospitals are not subject to income tax. The reverse is the case in regard to subscriptions from private individuals." The Committee suggests that a great impulse would be given to those who subscribe little or nothing to hospitals if subscribers could obtain relief from tax in respect of contributions to recognised medical charities. In short, it is proposed that subscriptions to hospitals should be treated by the Inland Revenue as insurance premiums are already treated, and within the limits set on them.

### THIS WEEK'S DRUG MARKET.

BUSINESS in the drug market has slackened down owing to the near approach of the Easter holidays; so far as the home trade is concerned business is almost at a standstill, but there is a constant stream of orders from overseas. As was expected, makers of mercurials have advanced their quotations in consequence of the upward movement in the price of quicksilver. Aspirin, phenacetin, and phenazone tend to move upward in price. The position of bromides is unchanged. Menthol and Japanese camphor continue very quiet. A consignment of buchu leaves has arrived from South Africa and will relieve the acute scarcity here; prices are extremely high. Citric acid continues very scarce and dear; tartaric acid is also scarce and the price is moving upwards; the same applies to cream of tartar; cod-liver oil is quiet, buyers holding off in the hope of lower prices.

